# Molecular genetics, correlation between genotype and phenotype of 65 Vietnames patients with congenital hyperinsulinism

**DOI:** 10.1186/1687-9856-2015-S1-P125

**Published:** 2015-04-28

**Authors:** Dang Anh Duong, Vu Chi Dung, Nguyen Phu Dat, Bui Phuong Thao, Can Thi Bich Ngoc, Nguyen Ngoc Khanh, Tran Minh Dien, Nguyen Thanh Liem, Sarah Flanagan, Sian Ellard

**Affiliations:** 1 National Hospital of Pediatrics, Hanoi, Vietnam; 2 Department of Molecular Genetics, Royal Devon & Exeter NHS Hospital. Peninsula Medical School, UK

## 

Hyperinsulinemic hypoglycemia (HH) is a consequence of unregulated insulin secretion by pancreatic β-cells. Congenital HH is caused by mutations in genes involved in regulation of insulin secretion (ABCC8, KCNJ11, GLUD1, CGK, HADH, SLC16A1, HNF4A and UCP2). Severe forms of congenital HH are caused by inactivating mutations in ABCC8 and KCNJ11, which encode the two components of the pancreatic β-cell ATP-sensitive potassium channel. Our aim is to identify mutations in the ABCC8 and KCNJ11, HNF4A and GLUD genes, and to describe genotype and phenotype correlations of Vietnamese children with congenital hyperinsulinism. A prospective study was conducted on 65 cases with congenital hyperinsulinism diagnosed and treated at the National Hospital of Pediatric from January 2007 to April 2014. Patients were selected by using inclusion criteria of Hussain K (2008). Mutations were identified in 32 cases (49.2%) including mutations of ABCC8 gene (28 cases; 43.1%), KCNJ11 (3 cases; 4.6%), HNF4A (1 case; 1.5%). 100% of cases with homozygous/compound heterozygous recessive mutations or one paternal dominant mutation of ABCC8 gene did not respond to diazoxide treatment and required 95% pancreatectomy. Molecular analysis using pancreas tissue after surgery from cases with one mutation of ABCC8 gene inherited from father confirmed focal lesion type. Other cases without identified mutations usually responded to diazoxide. In conclusions, children with congenital hyperinsulinism should be performed mutation analysis which helps in making diagnosis and treatment decision. Families of children with congenital hyperinsulinism should be given genetic counseling. Prenatal diagnosis should be performed as well as follow - up and treatment should be given to children with congenital hyperinsulinism immediately after birth.

